# Application of Ionic Liquids in Electrochemistry—Recent Advances

**DOI:** 10.3390/molecules25245812

**Published:** 2020-12-09

**Authors:** Gonçalo A. O. Tiago, Inês A. S. Matias, Ana P. C. Ribeiro, Luísa M. D. R. S. Martins

**Affiliations:** 1 Instituto de Tecnologia Química e Biológica, Av. da República, 2780-157 Oeiras, Portugal; goncalo.tiago@tecnico.ulisboa.pt; 2 Centro de Química Estrutural and Departamento de Engenharia Química, Instituto Superior Técnico, Universidade de Lisboa, Av. Rovisco Pais, 1049-001 Lisboa, Portugal; ines.matias@tecnico.ulisboa.pt

**Keywords:** ionic liquid, electrochemistry, electrolyte, electrodeposition, battery, cyclic voltammetry, tuneability

## Abstract

In this review, the roles of room temperature ionic liquids (RTILs) and RTIL based solvent systems as proposed alternatives for conventional organic electrolyte solutions are described. Ionic liquids are introduced as well as the relevant properties for their use in electrochemistry (reduction of ohmic losses), such as diffusive molecular motion and ionic conductivity. We have restricted ourselves to provide a survey on the latest, most representative developments and progress made in the use of ionic liquids as electrolytes, in particular achieved by the cyclic voltammetry technique. Thus, the present review comprises literature from 2015 onward covering the different aspects of RTILs, from the knowledge of these media to the use of their properties for electrochemical processes. Out of the scope of this review are heat transfer applications, medical or biological applications, and multiphasic reactions.

## 1. Introduction

Working safety is nowadays considered a more important issue than performance, and the delivery of safe and efficient materials for practical uses has been taken into account in the research and development (R&D) efforts. Thus, new features in materials are expected to appear in the immediate future, aiming to boost the transition to greener technologies towards meeting the goals of the 2030 EU Agenda for Sustainable Development [1] and resolving the ambitious challenges endorsed by the EU 2050 Green Deal [2]. Room temperature ionic liquids (RTILs), or simply ionic liquids (ILs), are an example of this new type of development, providing solutions for practical applications [3,4,5].

Ionic liquids are (mainly organic) salts exhibiting a very low melting temperature (liquids below 100 °C) and extremely low (negligible) vapor pressure [4]. Lately, they have received significant attention from the scientific community due to such unusual properties as liquids, being considered as suitable substitutes for volatile organic solvents (VOC, one of the major sources of waste in chemical synthesis) [6]. In fact, due to the unique physicochemical properties of ionic liquids the last decade has seen a remarkable boost on their application in several fields (e.g., chemistry, materials science or chemical engineering) with a significant high number of scientific contributions (ca. 68,057 publications in the period November 2010–2020, Figure 1).

Room temperature ionic liquids general physicochemical properties are summarized in Table 1.

Clearly, the physicochemical properties presented in Table 1 for RTILs are very different from those exhibited by ordinary molecular liquids. However, it is worth mentioning that every ionic liquid does not always show the above properties.

Ionic liquids are almost all times composed of organic ions, and therefore have unlimited structural variations in view of the easy preparation of many different components. The tuneability of combinations of cations and anions and the possibility to achieve modifications of the cation and/or the anion part offer access to ionic liquids with targeted properties. This has led to an exponential expansion of the number of multipurpose, interesting ILs known in recent years. Some of the most common ionic liquids components (cations and anions) are presented in Table 2.

Imidazolium-based ILs, such as 1–butyl–3–methyl–imidazolium [bmim] and 1–ethyl–3–methyl–imidazolium [emim], are among the most studied due to their stability within oxidative and reductive conditions, low viscosity, and ease of synthesis [8]. The cationic component of ILs has been varied to include pyridinium, ammonium, phosphonium, thiazolium, and triazolium species [5] (Table 2). In general, these cations have been combined with weakly coordinating anions, although not all weakly coordinating anions result in ILs [9]. Common examples include tetrafluoroborate, hexafluorophosphate, triflate, triflimide, and dicyanamide (Table 2). The first two have been explored the most and must be treated with the greatest caution as they are fairly readily hydrolysed to boric acid and phosphate, respectively [4]. Indeed, various phosphate and phosphinate anions (Table 2) have been employed bearing some advantages in RTILs [4,5].

Different potential applications of ionic liquids rely on their physicochemical properties, which vary based on the structures of the cation and anion used to assemble them (Table 2). A general understanding of these properties requires a detailed understanding of ionic liquid at the molecular level, namely the anion–cation interactions, which has been addressed in several previous reviews [5,10].

For instance, the ILs flame retardancy, based on the non-volatility inherent to ion conductive liquids, opens up the possibility for their application in the energy sector. In fact, ionic liquids are being developed for energy devices that need to use safe materials (avoiding accidental explosion or ignition) [7].

For electrochemical usage, the most important properties should be both electroconductivity and ion conductivity [11]. These are essentially the properties of advanced (and safe) electrolyte solutions that are critical to energy devices. For example, currently, electrochemical capacitors are limited in the cell voltage [12] due to the degradation of the applied electrolytes, based on organic solvents. Since the storable energy and power are dependent on the square of the cell voltage, it is worth investing in (safer) alternatives to the state-of-the-art electrolytes.

Electrolytes should have high ionic conductivity to minimize ohmic losses, high salt concentration to prevent starvation effects, a high electrochemical stability window in an appropriate temperature range, as well as high safety and low environmental impact.

Aqueous electrolytes exhibit very high ionic conductivities. However, their electrochemical stability window is very limited (the thermodynamic stability of water, at room temperature, is 1.23 V [13]), and therefore are not suitable to use at a wide temperature range or on a very small scale (due to the 100 °C boiling point of water) and are usually highly corrosive.

Non-aqueous common electrolytes (e.g., tetraethylammonium or triethylmethylammonium tetrafluoroborate salts in acetonitrile) also display very limited potential windows mainly due to the decomposition of the used solvent (e.g., the potential window of commercially available devices based on acetonitrile is 2.7 V [14]).

The use of ionic liquids as solvent free electrolytes might be one way to overcome the above potential window limitations. In fact, RTILs often display larger electrochemical stability windows (Table 3) [15,16] as compared to common non–aqueous electrolytes, concomitant with high thermal stability, non-flammability and in certain cases high conductivity.

RTILs exhibit a broad range of conductivities spanning from 0.1 to 20 mS cm^−1^ [17]. In general, higher conductivities are found for imidazolium based ILs in comparison with the ammonium ones. Many factors can affect their conductivity, such as viscosity, density, ion size, anionic charge delocalization, aggregations and ionic motions [18]. Strong ion-pair associations have been invoked in the case of bis(trifluoromethylsulfonyl)imide ([NTf_2_]^-^, Tf = triflate) based ILs, to understand their lower conductivity in comparison with tetrafluoroborate based ionic liquids [18].

The applicable electrochemical potential window of ILs can be determined using well-known electrochemical methods, such as cyclic voltammetry, and is typically found to be similar to or slightly larger than that found in conventional organic solvents, but much larger than that of aqueous electrolytes (Table 3). Imidazolium-based ILs display shorter electrochemical windows than the phosphonium ones indicating a higher electrochemical activity of the formers. In fact, by reduction, imidazolium ion can lead to the formation of N-heterocyclic carbenes [19]. Thus, the challenge is to design RTILs with a wide electrochemical window along with good electrical conductivity.

**Table 3:** Examples of electrochemical potential window of selected phosphonium- and imidazolium-based ionic liquids and standard aqueous and non-aqueous electrolytes.

IL	Solvent	Salt	Electrode	Potential Window/V	Reference
[P_2225_][NTf_2_]	-	-	Pt wire	6.3	[15]
[P_2228_][NTf_2_]				6.4	
[P_222(1O1)_][NTf_2_]				5.7	
[P_222(2O1)_][NTf_2_]				5.4	
[bmim][NTf_2_]			Carbon film	3	[16]
[bmim][NO_3_]				2.8	
[Pyr_14_][NTf_2_]			TiC-CDC	2.5	[20]
[Pyr_14_][NTf_2_]			AC	3.5	[21]
[EdMPN][NTf_2_]			Sucrose	±2.3	[22]
-	Acetone	[NEt_4_][ClO_4_], [NBu_4_][PF_6_], NaClO_4_	Pt wire	3.5	[23]
	CH_3_CN	[NEt_4_][ClO_4_], [NBu_4_][PF_6_], LiClO_4_		4	
	CH_2_Cl_2_	[NBu_4_][PF_6_], [NBu_4_][ClO_4_], [NBu_4_][X] (X = Cl, Br, F, I)		3.7	
	DMF	[NEt_4_][ClO_4_], [NBu_4_][PF_6_], LiCl, NaClO_4_		4.3	
	DMSO	[NEt_4_][ClO_4_], [NBu_4_][PF_6_]		3.3	
	THF	[NBu_4_][PF_6_], LiClO_4_, NaClO_4_		3.7	
	H_2_O	NaClO_4_,KNO_3_		2	

[P_2225_][NTf_2_] = triethyl-n-pentylphosphonium bis(trifluoromethylsulfonyl)amide; [P_222(1O1)_][NTf_2_] = triethyl(methoxymethyl)phosphonium bis(trifluoromethylsulfonyl)amide; [bmim][NTf_2_] = 1-butyl-3 methylimidazolium bis(trifluoromethylsulfonyl)imide; [Pyr_14_][NTf_2_] = 1-butyl-1-methylpyrrolidinium bis(trifluoromethanesulfonyl)imide; [EdMPN][NTf_2_] = ethyldimethylpropylammonium bis(trifluoromethylsulfonyl) imide.

Therefore, ionic liquids having a large electrochemical potential window and particularly unique physicochemical properties (e.g., negligibly small vapor pressure), constitute promising (and safer) candidates for the substitution of currently used electrolyte solutions based on organic molecular solvents. They have been applied as media for electrodeposition of metals [24], electrochemical biosensors [25], supercapacitors [26,27], batteries [28,29], and solar cells [30,31].

This review covers recent advances reported in the literature from 2015 to date, that have demonstrated a special focus on the application of ionic liquids as electrolytes for important electrochemical processes.

## 2. Imidazolium-Based Ionic Liquids

The imidazolium based ILs constitute the class of ionic liquids most used in electrochemical applications. The ones bearing two to four carbon atoms in the cation chain length, such as 1–butyl–3–methyl–imidazolium [bmim] or 1–ethyl–3–methyl–imidazolium [emim], respectively, are preferred, namely for electrodeposition processes.

Caporali et al. applied 1–Butyl–3–methyl–imidazolium bis(trifluoromethylsulfonyl)imide, [bmim][NTf_2_] (Tf = triflate) for the electrodeposition of a group of transition metals, such as silver, copper, cobalt, nickel, or zinc [32]. The metal was dissolved in such IL, under conditions suitable for industrial applications, i.e., an uncontrolled (moisture content) atmosphere, and electrochemically characterized by cyclic voltammetry and chronoamperometry.

Silver was present in [bmim][NTf_2_] in the form of uncoordinated or weakly coordinated Ag^+^ ions, and therefore represented the simplest electrochemical system. Homogeneous and crack-free silver coatings were potentiostatically obtained from this ionic liquid. The authors used a “dry” and a “wet” sample, were the water content was determined by Karl Fischer titration. The electroreduction of Ag^+^ was favoured in the “wet” system, but in both cases the reverse sweep showed a current crossover and a reduction peak that was dependent on the scan rate. Moreover, when they increased the scan rate, the peak potential value moved towards more negative values. Both of these observations are indicative that silver electroreduction follows a nucleation-growth mechanism. Thus, chronoamperometric measurements were carried out by stepwise variations of the potential of the working electrode from a value where no reduction of silver occurs to potentials sufficiently negative to induce the reduction process. In this manner, the electro-crystallization mechanism, by comparing experimental data with calculated values obtained from a theoretical model, was determined by the authors, that also compared their observations with previous published results obtained using other types of ionic liquids. They concluded that, in the “wet” system, the overpotential required to start the electroreduction is lower and the maximum current value is higher. This phenomenon was partially attributed to the enhanced Ag^+^ ion mobility due to the decreased viscosity of the electrochemical medium resulting from the presence of a larger amount of water. In the dry system, probably due to the lower mobility of the electroactive species, the effect of overpotential is less marked.

In contrast, copper-, cobalt-, and zinc-bearing systems were highly moisture sensitive. The majority of these metals were reduced at atmospheric pressure and at a scan rate of 100 mV.s^−1^. The authors pointed out that the addition of the metal salts to the hydrophobic [bmim][NTf_2_] considerably increases the ability of this IL to absorb water from the atmosphere and, therefore, the mild dehydrating experimental conditions employed assured only partial water removal. Therefore, the voltammogram recorded in the “dry” and “wet” system could not be directly compared, leading to a determination of the growth mechanism only in the “wet” systems. The authors concluded that the presence of large amounts of water changed the nature of the coordination species present in the solution, facilitating the electroreduction of the metal centres, and had also effects on the displayed colours of the samples. Nevertheless, metallic deposits were obtained, and their morphologies investigated as a function of the deposition potential and water content. Only in the case of the Ni-bearing IL did the authors report that it didn’t form electroactive species.

In this study, Caporali et al. [32] used very simple operating conditions that did not require a rigorously controlled atmosphere. This advantage made the process particularly easy for a potential upgrading for large low-cost applications.

**Figure 2:**
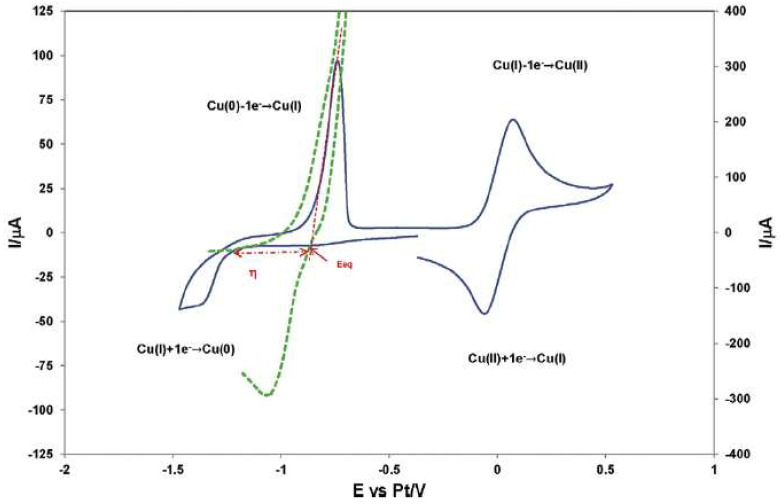
Cyclic voltammogram for Cu deposition on a Pt electrode exhibiting a nucleation overpotential (green); Cyclic voltammogram obtained on a Pt electrode in the [bmim]Cl without Cu(I) (blue). Reproduced with permission from [33], copyright 2016, Elsevier.

The IL 1-butyl-3-methyl-imidazolium chloride, [bmim]Cl, was used as electrolyte in the study of the redox properties of Cu(I) and Cu(II) ions, showing that the Cu(I) ion can be oxidized to Cu(II) and reduced to Cu(0), and that Cu(II) can be reduced to Cu(I) by the metallic Cu(0) [33]. These electrochemical processes are depicted in Figure 2.

According to the authors, the experimental voltammograms suggested the presence of a nucleation phenomenon (Figure 2), but mainly in a qualitative way. The electrodeposition of copper occurs, and the explanation given is that the formation of stable Cu nuclei on an inert surface requires a potential more negative than the reduction of Cu(I) on a copper surface. Chronoamperometry was also performed to understand the nucleation/growth process of copper on a Pt wire and a Pt disc electrode.

The applications of rare earth metals in technological devices such as cell phones, and other electronic devices, as well as in permanent magnets leads to a pressing need to find a way to recycle them.

**Figure 3:**
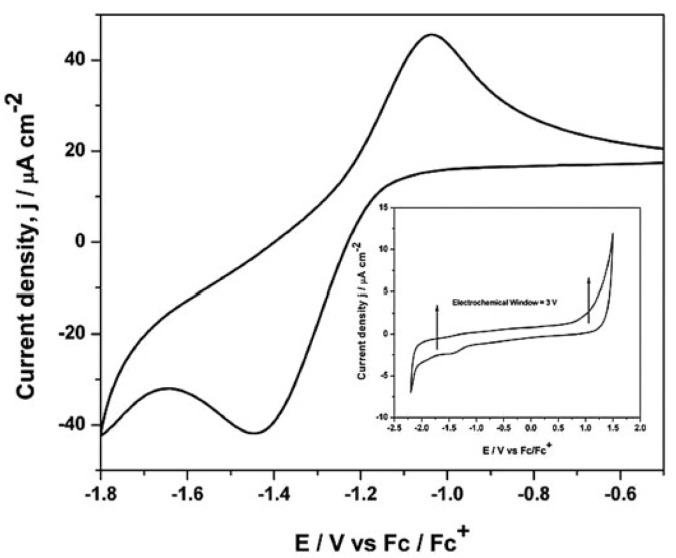
Cyclic voltammogram of 50 mM Eu(III) ions in [bmim][NTf_2_] at 20mV/s. Inset: Cyclic voltammogram of [bmim][NTf_2_] at a glassy carbon electrode at 25 °C. Reproduced with permission from [34], copyright 2015, Wiley.

Gupta et al. [34], studied, by cyclic voltammetry and chronoamperometry at a glassy carbon electrode, the electrochemical properties of europium(III) aiming at to understand the oxidation state, coordination geometry, and physicochemical behaviour of the Eu(III) complex bearing di-hexyl-*N*,*N*-diethylcarbamoylmethylphosphonate (DHDECMP) ligand (as complexing extractant) in [bmim][NTf_2_]. The reduction of Eu(III) to Eu(II) in [bmim][NTf_2_] has shown a quasi-reversible reduction, at a peak potential of −1.44 V vs. Fc/Fc^+^ (Fc = ferrocene), and was controlled by diffusion and charge transfer kinetics (Figure 3).

The cyclic voltammogram of [bmim][NTf_2_], recorded at a glassy carbon working electrode, is shown in the inset of Figure 3. The reduction of the [bmim]^+^ cation occurs at a potential of −2.0 V (vs. Fc/Fc^+^), and the oxidation of the [NTf_2_]^–^ anion occurs at 1.0 V (vs. Fc/Fc^+^), allowing to detect the reduction of Eu(III) to Eu(II). Photoluminescence spectroscopy confirmed that the symmetry around the europium ions at Eu^3+^–DHDECMP in the [bmim][NTf_2_] was relatively low. By fluorescence lifetime measurements, the authors also determined that the number of water molecules in the inner sphere is six for non-complexed europium ions, and practically no water molecules was retained in the presence of the complexing extractant DHDECMP.

**Figure 4:**
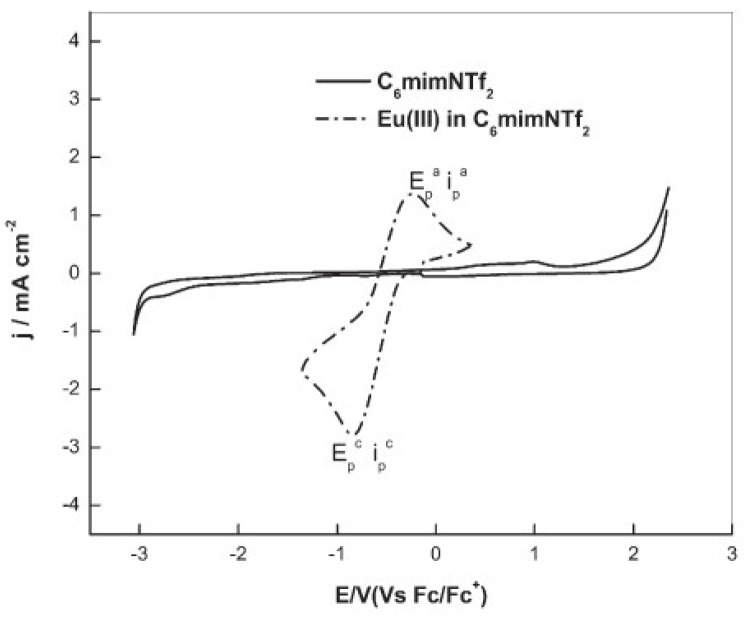
Cyclic voltammetry of [hmim][NTf_2_] (—) and Eu(III) in [hmim][NTf_2_] (----) recorded at glassy carbon electrode. [Eu(III)] = 100 mM, scan rate = 100 mV/s, T = 373 K. Reproduced with permission of ref. [35], copyright, 2015, Springer.

An IL with a higher cation chain length, [hmim][NTf_2_] (hmim = 1–hexyl–3–methyl–imidazolium), was applied in the study of the redox properties of europium(III) for the recovery of europium by an extraction–electrodeposition (EX-EL) procedure [35]. The cyclic voltammogram of Eu(III) in [hmim][NTf_2_], recorded at a glassy carbon electrode, exhibited a prominent quasi-reversible reduction wave occurring at the onset of −0.25 V vs. Fc/Fc^+^ (Fc = ferrocene), culminating in a peak at −0.84 V vs. Fc/Fc^+^ assigned to the reduction of Eu(III) to Eu(II), as shown in Figure 4.

**Figure 5:**
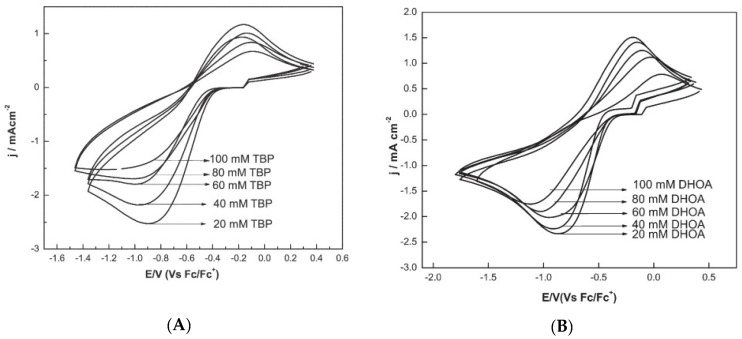
Cyclic voltammograms of Eu(III) (100 mM) recorded at glassy carbon electrode at a scan rate of 100 mV s^−1^ and at 373 K, in (**A**) TBP/[hmim][NTf_2_] or (**B**) in DHOA/[hmim][NTf_2_]. Reproduced with permission of ref. [35], copyright, 2015, Springer.

The cathodic peak current was lowered, and the peak potential shifted cathodically in the presence of tri-n-butyl phosphate (TBP) and N,N-dihexyloctanamide (DHOA) as ligands, due to the co-ordination of Eu(III) to such species in the ionic liquid medium (Figure 5). Moreover, the presence of TBP in the ionic liquid medium shifts the Eu(III) to Eu(II) peak potential to more negative values as compared to DHOA (compare Figure 5A,B).

The stability constants of Eu(III)–ligand complex showed that the stability of Eu-DHOA complex in ionic liquid medium was lower than that of the Eu-TBP complex, and therefore the use of DHOA ligand for facilitating the electrodeposition of europium from the ionic liquid during the recovery of europium by extraction–electrodeposition (EX-EL) procedure would be the best option.

**Figure 6:**
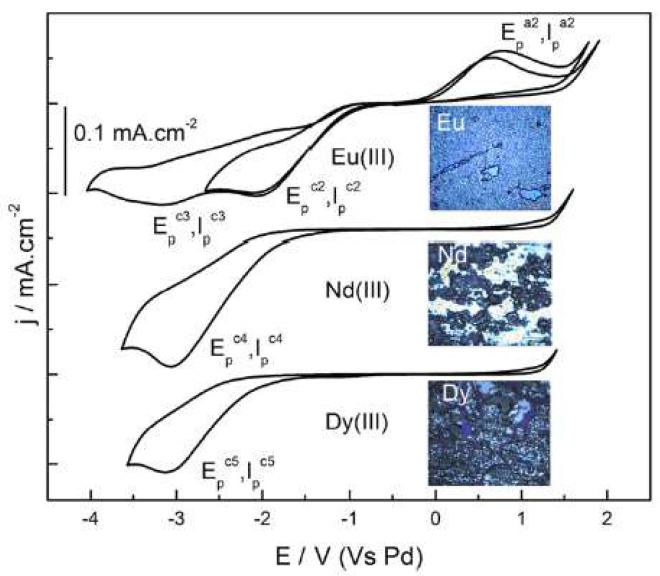
Cyclic voltammograms of 100 mM [Ln(NTf_2_)_3_] in DHOA medium recorded at glassy carbon working electrode at the scan rate of 50 mV s^−1^ at 353 K. Insets show the photographs of the electrodeposits obtained at the working electrode after electrolysis of 100 mM Ln^3+^ in the system DHOA/[bmim][NTf_2_] at −3.0 V for about 2 h. Reproduced with permission from [36], copyright 2020, Elsevier.

Krishna et al. [36], attached the neutral ligand DHOA to [bmim][NTf_2_] to study the electrodeposition of a series of lanthanides (Ln), such as europium, neodymium, or dysprosium. The reduction of Eu^3+^, Nd^3+^, and Dy^3+^ to their respective metallic forms was successfully achieved (Figure 6).

To enhance their solubility, the investigation of the electrochemical behaviour of the lanthanide(III) ion present in neutral ligand-ionic liquid (NLIL) was performed by cyclic voltammetry. Figure 6 shows that the there is a two-step reduction observed for Eu^3+^, whereas the NLIL containing Nd^3+^ and Dy^3+^ underwent a single step 3-electron transfer reduction at the cathodic potential of −3.0 V. The authors also confirmed that the anodic waves, corresponding to the oxidation of Nd^0^ or Dy^0^ to higher oxidation states, were not observed during the performed reversed scans.

Sengupta et al., [37] used [bmim][NTf_2_] to report a first-ever cyclic voltammetric electrochemical characterization of neptunium(IV) complexes bearing the diglycolamide ligand. Np(IV) was found to undergo mono-electron exchange reactions. The activation energy values were deduced using the Arrhenius equation and thermodynamic parameters were derived using linear regression of the data. The redox reactions of the Np(IV) complexes were exothermic. In a further study, the authors [38] detected, by cyclic voltammetry, a different electrochemical behaviour for neptunium(IV) bearing diphenyl-N,N-diisobutylcarbamoyl-methylphosphine oxide in [bmim][NTf_2_] (CMPO-NTf_2_): for the redox couples involving complexes of Np(IV) with CMPO or CMPO-NTf_2_, the enthalpy values were positive or endothermic.

**Figure 7:**
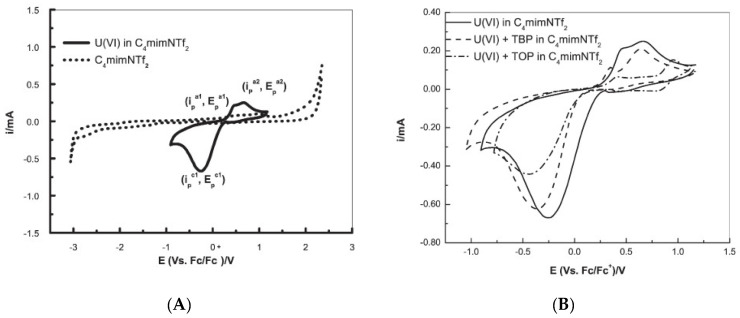
Cyclic voltammograms recorded at a glassy carbon electrode ([U(VI)] = 100 mM, scan rate = 100 mV s^−1^, T = 373 K) of (**A**) [bmim][NTf_2_] (----) and U(VI) in [bmim][NTf_2_] (—); and (**B**) U(VI) in [bmim][NTf_2_], in TBP/[bmim][NTf_2_] and in TOP/[bmim][NTf_2_]. Reproduced with permission from [39], copyright 2016, Elsevier.

Later, Rama et al., [39] used [bmim][NTf_2_] to investigate the electrochemistry performance of U(VI). The cyclic voltammogram of U(VI) in [bmim][NTf_2_]*,* recorded at a glassy carbon working electrode at the scan rate of 100 mV/s at 373 K, exhibited a prominent quasi-reversible reduction of U(VI) to U(V) (Figure 7), where the cathodic peak potential was shifted anodically and the cathodic peak current increased with the increase of temperature. The presence of tri-n-octyl phosphate (TOP) or tri-n-butyl phosphate (TBP, a reference extractant) ligands shifted the cathodic peak to more negative potentials due to the formation of a U(VI) complex (compare Figure 7A,B).

One year later, Krishna’ group extended the study of the redox properties of U(VI) by using [bmim][Cl], which increased the cathodic current density and consequently the U(VI) reduction was facilitated [40].

The above work was followed by the use of imidazolium-based IL bearing the dicyanamide anion [bmim][DCA], by the same researchers [41], to study the electrochemical behaviour of UO_2_^2+^. The cathodic peaks corresponding to the reduction of UO_2_^2+^ to UO_2_^+^ and then to UO_2_ were clearly detected, indicating that [bmim][DCA] is a good solvent for this lanthanide cation.

Aiming at to accelerate the energy transition (urgent to be adopted in order to reduce anthropogenic causes for climate changes), an intense research is being developed in electrochemical processes of energy conversion and storage, through the design of new materials, namely ionic liquids, which would be able to increase the energetic efficiency of important processes.

The suitability of room temperature ionic liquids as solvents for redox flow batteries (RFBs) containing metal complexes was investigated by Ejigu et al. [42], using metal acetylacetonate (acac) complexes [M(acac)_3_], (M = Mn, Cr, or V) in imidazolium based RTILs. They detected, by cyclic voltammetry at a glassy carbon electrode, an irreversible behaviour for [Mn(acac)_3_] and [Cr(acac)_3_] in 1-ethyl-3-methylimidazolium bis(trifluoromethanesulfonyl)imide [emim][NTf_2_], but the rate of the Mn^2+^/Mn^3+^ reaction increased when Au electrodes were used.

**Figure 8:**
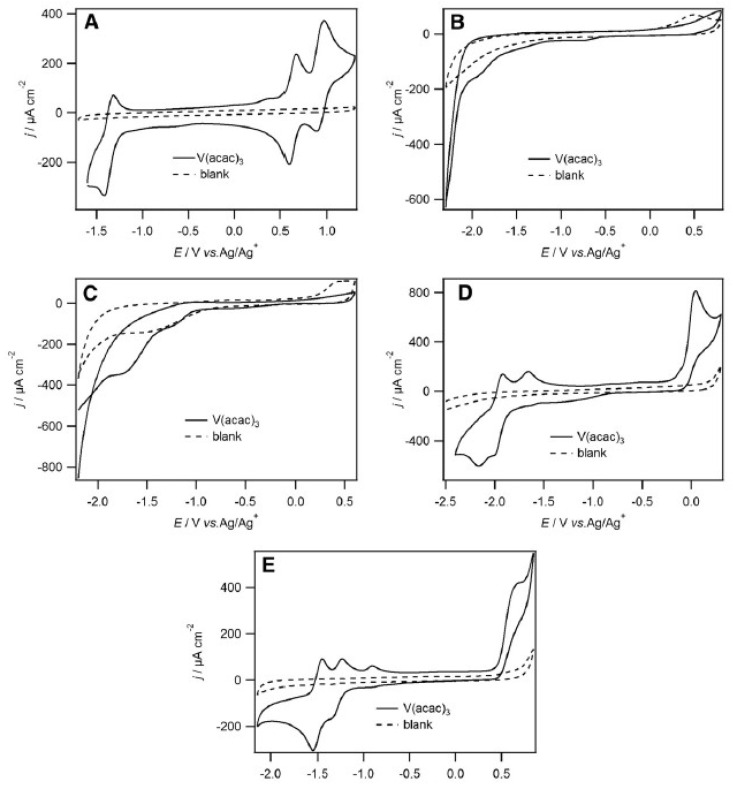
2nd cycles of cyclic voltammograms recorded at a 5 mm diameter glassy carbon disk electrode at 50 mV/s of (**A**) [V(acac)_3_] (10 mM) in [emim][NTf_2_], (**B**) [V(acac)_3_] (11 mM) in [bmim][BF_4_] (**C**) [V(acac)_3_] (12 mM) in [bmim][PF_6_], (**D**) [V(acac)_3_] (12 mM) in [emim][N(CN)_2_] and (**E**) [V(acac)_3_] (20 mM) in [emim][EtSO_4_]. All cyclic voltammograms began at the negative potential limit. Reproduced with permission from [42], copyright 2020, Springer.

The authors also observed that V^2+^/V^3+^, V^3+^/V^4+^ and V^4+^/V^5+^ redox couples were quasi-reversible in 1-ethyl-3-methylimidazolium bis(trifluoromethanesulfonyl)imide [emim][NTf_2_], by cyclic voltammetry at glassy carbon electrode (with a high columbic efficiency of 72%). Moreover, among the following RTILs studied, namely [bmim][BF_4_], [bmim][PF_6_], [emim][N(CN)_2_], and [emim][EtSO_4_], [EtSO_4_]^−^ is the ethylsulfate anion (Figure 8), and only [emim][NTf_2_] stabilized the various V^n+^ species (Figure 8A) [42]. In fact, in Figure 8A, we can see clearly three well-defined redox couples centred at −1.37, 0.63, and 0.90 V corresponding to V^2+^/V^3+^, V^3+^/V^4+^ and V^4+^/V^5+^ oxidations, respectively. Also, the potential difference, ΔE, between the V^2+^/V^3+^, V^3+^/V^4+^ and V^4+^/V^5+^ couples is 2.0 V, which is comparable to several organic solvents. Such a decrease in ΔE for consecutive redox waves upon changing solvents from acetonitrile to RTILs seems to demonstrate that the RTIL are more basic than most of the common organic solvents. Figure 8B,C show that the [V(acac)_3_] redox couples were not clearly discernible in [bmim][BF_4_] and [bmim][PF_6_]. In [emim][N(CN)_2_] and [emim][EtSO_4_], the redox couple at positive potentials was chemically irreversible (Figure 8D,E). Interesting is in the particular case of [emim][N(CN)_2_], were a cathodic, return peak did appear as the scan rate increased and the ratio of the cathodic to anodic peak currents, also increased. This suggests that a following chemical reaction occurred after oxidation.

In addition, they observed that the use of [bmim][NTf_2_] yields almost identical voltammetry to that observed in [[emim][NTf_2_], demonstrating that the RTILs anions played the most significant roles in stabilizing the V^n+^ species. They also discussed the importance of diffusion coefficients. The low diffusion coefficients measured, which shown that [emim][NTf_2_] are an order of magnitude lower than in acetonitrile, allied with the relatively high viscosity of RTILs can suggest that the RFBs containing [emim][NTf_2_] as the electrolyte could face mass transport issues.

Balo et al. [43] applied 1-ethyl-3-methylimidazolium bis(trifluoromethylsulfonyl)imide, [emim][NTf_2_], for the recharge of lithium batteries, with promising results. They formed a gel polymer electrolyte (GPE) for high performance lithium polymer batteries (LPBs). The gel is based on the polymer polyethylene oxide and the lithium bis(trifluoromethylsulfonyl)imide salt. By cyclic voltammetric studies, the authors discovered that the gel exhibits promising characteristics suitable for application in LPBs. The GPEs show high thermal stability, high ionic conductivity, high lithium transference number and high electrochemical stability window. One year later, Prasanna and collaborators [44] reported the use of [bmim][NTf_2_] incorporated in a polymeric matrix, containing polyvinyl chloride (PVC) and poly(ethyl methacrylate) (PEMA), for the electrochemical study of zinc ion conducting gel polymer electrolytes (GPEs). The prepared films of gel polymer membranes were fully characterized utilizing complex impedance spectroscopy, differential scanning calorimetry thermogravimetric, and cyclic voltammetry analyses. A similar trend was observed for the dielectric constant and ionic conductivity with the increase of [emim][NTf_2_] concentration.

**Figure 9:**
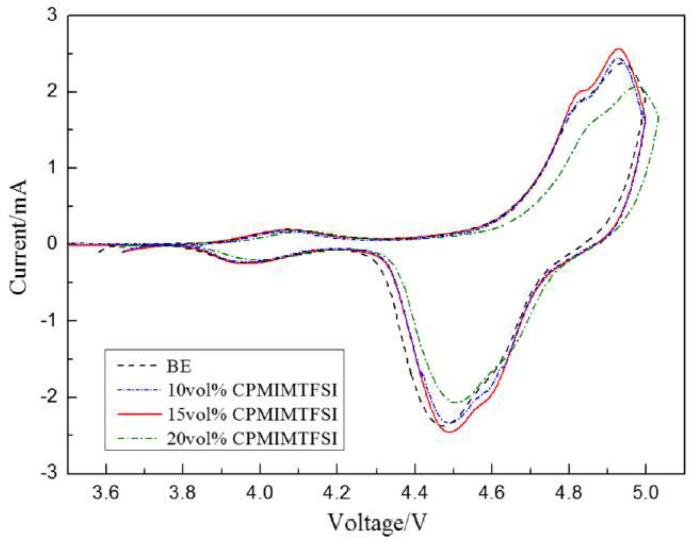
Cyclic voltammograms of LiNi_0.5_Mn_1.5_O_4_/Li cells after 30 cycles with different concentrations of [cpmim][NTf_2_] at 0.1 mV s^−1^. Reproduced with permission from [45], copyright 2020, Springer.

Zhang group reported that the use of 1-cyanopropyl-3-methylimidazolium bis(trifluoromethanesulfonyl)imide, [cpmim][NTf_2_], as additive in electrolyte for high-voltage lithium-ion batteries (LIBs), sharply increasing the discharge capability of these batteries [45]. Cyclic voltammograms of the LiNi_0.5_Mn_1.5_O_4_/Li system are presented in Figure 9, displaying two redox peaks on each curve.

The oxidation peaks were mainly associated to the redox reactions undergone by the transition metals present at LiNi_0.5_Mn_1.5_O_4_/Li. Moreover, the electrolyte with 15 vol.% of [cpmim][NTf_2_] had good integrity and large current density. The redox peaks of 20 vol.% of [cpmim][NTf_2_] presented lower current intensity than the others, and the oxidation peak was anodically shifted. The authors presented two possible reasons for this shift. One was that the high viscosity of 20 vol.% of [cpmim][NTf_2_] increased the internal resistance and the other reason may be related to the high concentration of ionic liquids that led to an enrichment phenomenon on the surface of cathode due to the charge of ionic liquid. As a consequence, the surface structure of the cathode was changed, and thus the peak had a slight shift. Even so, the authors concluded that the mixed electrolyte with [cpmim][NTf_2_] is promising to be used in the application of Li-ion batteries.

Recently, the chloroaluminate imidazolium ionic liquid AlCl_3_/[emim][Cl] was used to test the performance of titanium dioxide (TiO_2_) as electrode in rechargeable aluminum-ion batteries. TiO_2_ could be a promising electrode material for aluminum-ion battery but it is still quite understudied. Das et al., [46] demonstrate the rechargeability of an Al-ion cell with anatase TiO_2_ as cathode in the chloroaluminate ionic liquid electrolyte. The cell of Al-TiO_2_ was proven to have an excellent long-term stability. Also, the electroactive nature of TiO_2_ in the chloroaluminate electrolyte was studied both by cyclic voltammetry and galvanostatic cycling studies. The authors observed the existence of a synergistic effect of the current collector in improving the long-term stability of Al-TiO_2_ cell.

In the same year, Huang and collaborators [47] also demonstrated that the use of pure 1-ethyl-3-methylimidazolium trifluoromethanesulfonate IL, [emim][OTf], as an ionic liquid electrolyte is a good choice for the design of a dual-graphite battery.

Besides the usage of ILs themselves as electrolytes, some authors have applied them as electrolyte additives. For example, very recently [48], [emim][NTf_2_] was studied by cyclic voltammetry for usage as possible additive to the electrolyte in sodium ion batteries Electrochemical cycling of a hard carbon electrode reveals that the [NTf_2_]^-^ anion, mainly coming from the added ionic liquid, can introduce new peaks in the cyclic voltammograms or new steps in the galvanostatic discharge. The products of such degradation can make an interface which determines the cycling properties.

One very interesting work involving the application of ionic liquids as electrolyte additives was developed by Wong et al. [49], who studied the concentration of [emim][BF_4_] in an electrolyte to optimize the capacitive performance of high-energy density graphene supercapacitors. The authors reported that the electrolyte viscosity increased exponentially with the increase of IL concentration, while the ionic conductivity decreased with an increase in IL concentration. They also found a strong dependence of the specific capacitance not only on the electrolyte viscosity and ionic conductivity, but also in the maximum working voltage (MWV) where the electrode specific capacitance increases. The neat IL (i.e., [emim][BF_4_]) offered the largest specific capacitance and energy density among all IL concentrations for graphene-based electric double-layer capacitors (EDLC), despite having the highest viscosity and the lowest ionic conductivity. The large specific capacitance and energy density was also due to the largest MWV offered by this neat ILs. Another advantage reported by the authors was that, if the EDLC does not require to operate at the electrochemical stability window of the IL, diluted IL electrolytes with an optimized concentration/IL viscosity can be used instead. To sum up, they concluded that the concentration of IL electrolyte should be optimized according to the working voltage required.

[Emim][N(Tf_2_)] was also used for studying the electrochemistry of I^−^, I_2_ and ICl, together with [bmim][Cl], due to the integral role of the I^−^/I^3−^ couple in dye-sensitized solar cell technology [50]. Bentley et al., used the cyclic voltammetric technique at a platinum macrodisk electrode in neat ILs, as well as a binary mixture of [bmim]Cl and [emim][N(Tf_2_)]. The neutral (I_2_) and positive (I^+^) oxidation states of iodine are known to have strongly electrophilic behavior, and thus the I^−^/I_2_/I^+^ redox processes are sensitive to the presence of nucleophilic chloride or bromide. These anions are both commonly present as impurities in non haloaluminate RTILs. In the absence of chloride (e.g., in neat [emim][N(Tf_2_)], I^−^ was oxidized in an overall one electron per iodide ion process to I_2_ via an I_3_^−^ intermediate, gave rise to two resolved I^−^/I_3_^−^ and I_3_^−^/I_2_ processes. When Cl^-^ and I^−^ were present, even in low concentrations (below 30 mM), an additional oxidation process appears at potentials less positive than the I_3_^−^/I_2_ process. This corresponded, according to the authors, to the oxidation of I_3_^−^ to the inter-halide complex anion [ICl_2_]^−^ in an overall two electrons per iodide ion process.

Also, the electrochemistry of I^−^, I_2_, and ICl has been investigated by cyclic voltammetry in a binary IL mixture composed of [bmim]Cl and [emim][NTf_2_], using the same experimental conditions. The authors studied the effect of the presence of a large excess of Cl^−^ ([I^−^] ≈ 10 mM and [Cl^−^] ≈ 3.7 M) and observed that I^−^ was oxidized in an overall two electrons per iodide ion process to [ICl_2_]^−^ via an [I_2_Cl]^−^ intermediate. Bentley and coworkers concluded that in the I^−^/I_2_/I^+^ processes, in non haloaluminate ILs, a complicated interplay between multiple electron transfer pathways and homogeneous chemical reactions is present, which may not be at equilibrium on the voltametric time scale.

A mixture of the IL [bmim][BF_4_] with acetonitrile as electrolyte was used by Lebedeva et al., to investigate the electrochemical behavior of polyaniline composites, and the author proposed a mechanism involving [bmim]^+^ as protons source [51].

The search for safer and more sustainable operation conditions has been also pursued in areas such as the mitigation of the greenhouse gas carbon dioxide (a major driver of climate change) through its transformation into value-added products. In fact, CO_2_ conversion into valuable chemicals would be the most promising way to reduce CO_2_ emissions, making it a part of the solution as carbon feedstock. Moreover, such CO_2_ transformation would preferably occur in conditions that allow to avoid the use of volatile and toxic solvents or catalysts (according to the growing demand for eco-friendly synthetic methodologies), as well as of any supporting electrolyte (for a very easy workup of the reaction mixture).

**Figure 10:**
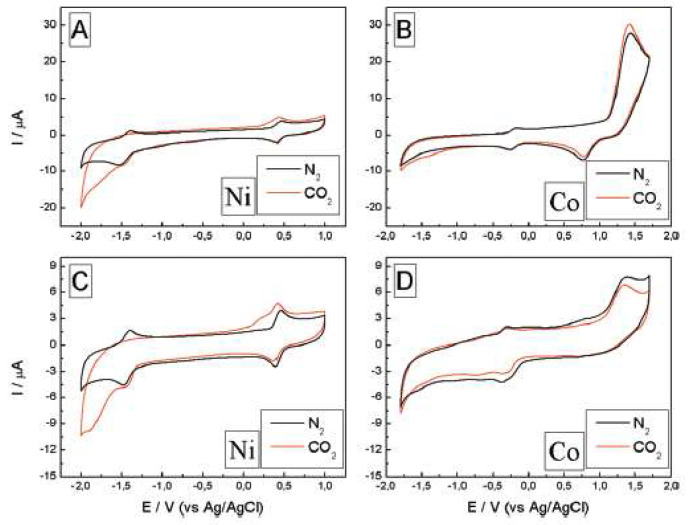
Electrochemical behavior of the system in [bmim][BF_4_] (**A**,**B**) and [bmim][NTf_2_] (**C**,**D**), [Ni(cyclam)Cl_2_] and [Co(cyclam)Cl_2_]Cl at 9.7 mM and 2 mM, respectively, both saturated with N_2_ (black line) and CO_2_ (red line), at 100 mV/s. Reproduced with permission from [52], copyright 2017, Elsevier.

Honores et al. [52] used [bmim][BF_4_] and [bmim][NTf_2_] to study the electrochemical reduction of carbon dioxide in the presence of cyclam Ni^2+^ and Co^3+^ catalysts (Figure 10).

[Ni(cyclam)Cl_2_] exhibited a better performance than [Co(cyclam)Cl_3_]. Moreover, the solvent had a major impact on the catalytic performance of the systems: the hydrophilic ionic liquid [bmim][BF_4_] promoted the catalytic activity, while [bmim][NTf_2_] led to very low values of TON.

A study using an ionic liquid bearing the triflate anion, [bmim][OTf], was also used to characterize the cation bismuth/[bmim]^+^, under conditions for CO_2_ reduction [53].

Ratschmeier et al., [54] studied the CO_2_ reduction reactions (CO_2_RR) at Pt electrodes in four ionic liquids. These electrolytes were 1-ethyl-3-methylimidazolium dicyanamide, [emim][DCA], [emim][BF_4_], [bmim][BF_4_], and 1-ocytl-3-methylimidazolium tetrafluoroborate, [omim][BF_4_]. It was found that water played an important role in CO_2_ reduction at a Pt electrode, demonstrating that the formation of an imidazolium carboxylic acid intermediate occurs at electrode potentials of −0.4 V. The authors also reported evidence of the formation of CO. As a consequence, the presence of CO led to deactivation of the Pt surface and to a decrease in reduction currents.

On the other hand, [bmim][NTf_2_] was used for the study of adsorption and oxidation of CO. In such work, beyond the CO oxidation, the oxidation of the [NTf_2_]^-^ anion and the release of a proton were observed [55].

The detection of gases has also been explored by cyclic voltammetry in ionic liquids. Yu’s group [56] used [bmim][PF_6_] for this purpose. They modified this IL with a nickel oxide with reduced graphene oxide to increase the sensitivity to detect O_2_.

**Figure 11:**
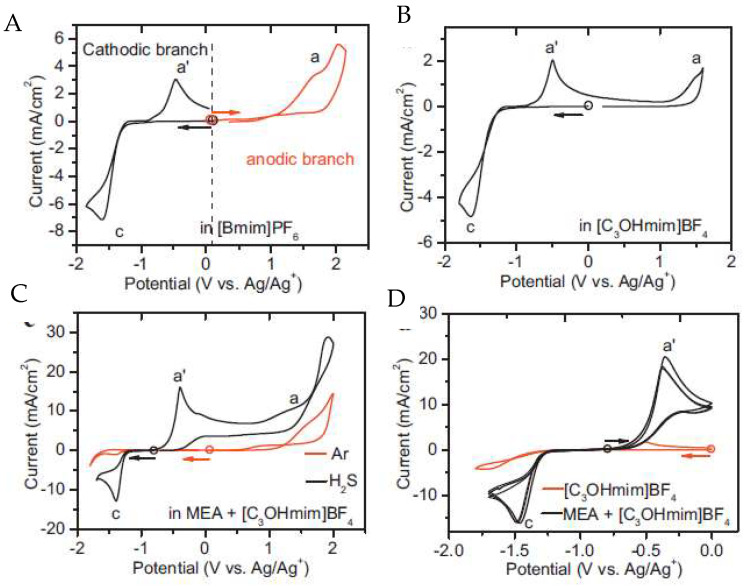
Cyclic voltammograms of H_2_S at the Pt microdisk electrode (diameter = 100 μm) in different electrolytes. (**A**) is [bmim][BF_4_]; (**B**) is [C_3_OHmim][BF_4_]; (**C**) is [C_3_OHmim][BF_4_] with MEA and atmospheric gases and (**D**) is a comparison of cyclovoltamograms between [C_3_OHmim][BF_4_] and [C_3_OHmim][BF_4_] with MEA. Scan rate: 50 mV s^−1^; the circle indicates the open cell potential (OCP) of each system, and the arrow indicates the start direction of the potential scan from OCP. Reproduced with permission from [57], copyright 2018, Elsevier.

Huang and collaborators [57] used the functionalized imidazolium IL 1-hydropropyl-3-methylimidazolium tetrafluoroborate, [C_3_OHmim][BF_4_], for the detection of H_2_S. Due to the high absolute values of potential needed to oxidize or reduce H_2_S, monoethanolamine (MEA) was added to facilitate its detection (Figure 11).

In both [bmim][BF_4_] (Figure 11A) and [C_3_OHmim][BF_4_] (Figure 11B) media, the oxidation/reduction of H_2_S occurred at a very high/low potential, close to the upper/lower limit of the electrochemical window of the RTILs. This behaviour led to the conclusion that they were unsuitable for H_2_S sensing. Moreover, the addition of MEA to [C_3_OHmim][BF_4_] electrolyte (Figure 11D increases the solubility of H_2_S through chemical absorption, releasing electroactive HS-ions, which subsequently led to an additional anodic response at a potential appropriate for the detection of H_2_S. As a final remark, in this study it was possible to conclude that the detection is not affected by ambient gases such as CO_2_ or SO_2_.

Recently, other functionalized imidazolium ILs were applied in electrochemistry. For example, 1-(2′,3′-dihydroxypropyl)-3-methylimidazolium hydroxide, [dhpmim][OH], provided a good capacitance to nickel oxide nanosheets as electrodes [58]. Further, the electrochemical stability windows of a series of sulfonic-functionalized ILs with trifluoroacetate anion in molecular solvents were investigated, presenting wide potential windows in acetonitrile [59].

**Figure 12:**
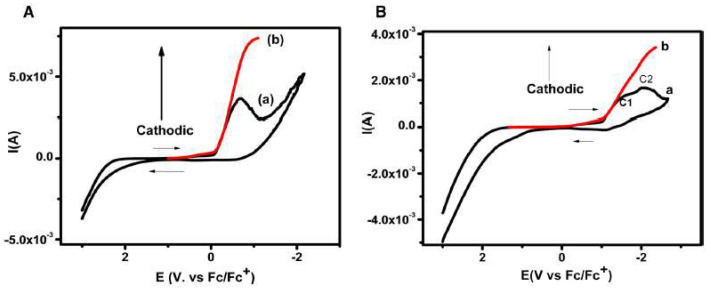
(**A**) Cyclic voltammogram of 5 M thymine at 400 mV/s in [bmim][BF_4_] depicting; (**a**) original cyclic voltammogram, (**b**) convoluted cyclic voltammogram. (**B**) Cyclic voltammogram recorded at 400 mV s^−1^ for 5 mM thymidine in [bmim][BF_4_], (**a**) original (**b**) convoluted. Reproduced with permission from [60], copyright 2019, Elsevier.

In a completely different research area, the electrochemical behaviour of the nucleobases thymine and thymidine was investigated in [bmim][BF_4_], observing one- and two-electron reduction peaks, respectively [60], as it is shown in Figure 12. The chronoamperometric fit and the cyclic voltammetry at different scan rates confirmed an irreversible one-electron transfer in both thymine and thymidine.

The corrected cyclic voltammogram of thymine in [bmim][BF_4_] at 400 mV s^−1^ (Figure 12A), exhibits the reduction peak at −0.67 V vs. Fc/Fc^+^ redox couple. The authors were able to elucidate the electrochemical reductive mechanism involved in the reduction of thymine and thymidine in [bmim][BF_4_] by convolutive semi-integral analysis of the cyclic voltametric data produced. The electrochemical reduction of thymine followed a one-electron transfer at the electrode surface resulting in the formation of electron adducts of thymine at the cathode peak. This mechanism is supported by theoretical simulations of the variation of bond dissociation free energy via the concerted sticky dissociative model [60].

From the examples presented above, it is clear that imidazolium based ILs are very promising electrochemical electrolyte media, mainly due to their electrochemical stability and versatility for numerous applications.

## 3. Ammonium-, Pyrrolidinium-, Phosphonium- and Sulfonium-Based ILs

In this section we will present progresses in electrochemistry using ILs other than imidazolium-based ones.

Out of the several ILs, the quaternary ammonium salts are an economically advantageous class of ionic liquids used in different applications as they exhibit better thermal and chemical stability compared to pyridinium- and imidazolium-based compounds.

**Figure 13:**
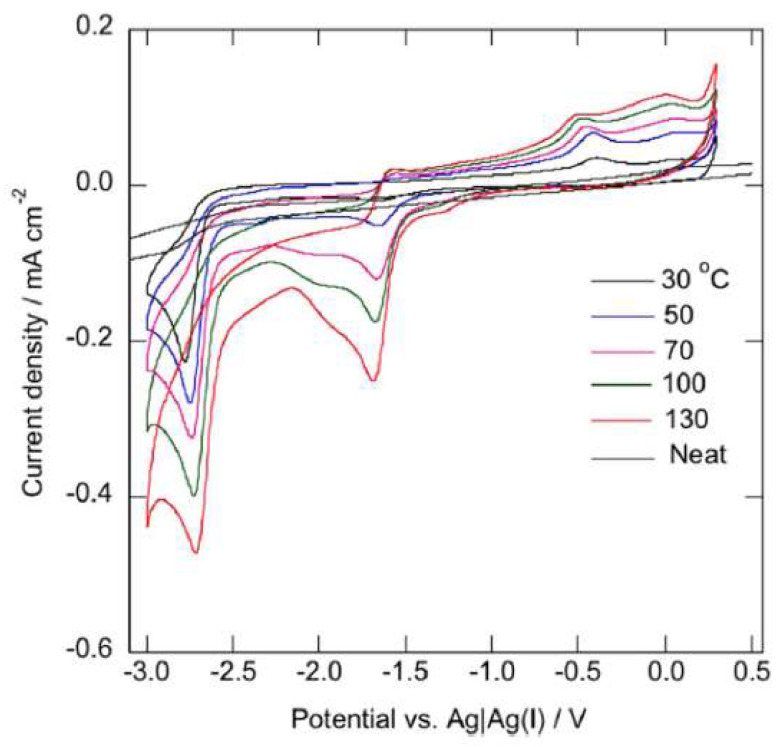
Cyclic voltammograms of a glassy carbon electrode in [N_1116_][NTf_2_] containing 5 mM [Pt(acac)_2_] at various temperatures. Scan rate: 50 mV/s. Reproduced with permission from [61], copyright 2016, RSC.

Sultana et al. [61] used *N*-trimethyl-*N*-hexylammonium bis(trifluoromethylsulfonyl)amide, [N_1116_][NTf_2_], to study the electrochemical behaviour of an acetylacetonate (acac) platinum(II) complex, [Pt(acac)_2_], as function of temperature (Figure 13). A cathodic peak corresponding to the reduction of Pt(II) to Pt(0) and consequent electrodeposition of this metal on the electrode was found to depend on the diffusion of Pt(acac)_2_ in the [N_1116_][NTf_2_] IL.

**Figure 14:**
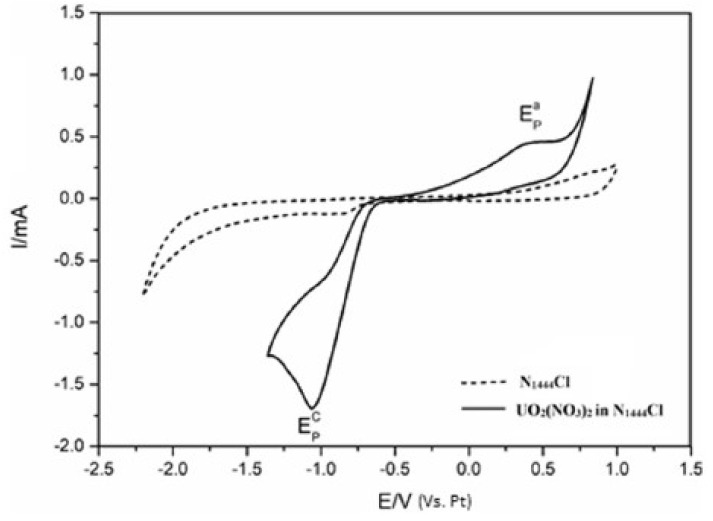
Cyclic voltammogram of [N_1444_]Cl and UO_2_^2+^ in [N_1444_]Cl recorded at glassy carbon electrode. [UO_2_^2+^] = 0.07 M, temperature = 383 K, scan rate = 100 mV s^−1^. Reproduced with permission from [62], copyright 2020, Elsevier.

The ammonium-based ionic liquid tri-n-butylmethylammonium chloride, [N_1444_]Cl, was selected by Bhujbal et al. [62] to study the electrochemical behaviour of uranyl ion, UO_2_^2+^ (Figure 14). An irreversible reduction of U(VI) to U(IV) was observed, resulting in the electrodeposition of uranium oxide (UO_2_) over the glassy carbon working electrode.

As depicted in Figure 14, [N_1444_]Cl exhibits the electrochemical window of 2.8 V with the cation reduction potential of −1.9 V and the anion oxidation potential of +0.9 V. The use of [N_1444_]Cl as electrolyte for the UO_2_^2+^/UO_2_ reduction couple allowed a fast (40 min) deposit of a considerable amount of uranium in the form of uranium dioxide.

Arkhipova and coworkers [63] employed tetraethylammonium bis (trifluoromethylsulfonyl)imide, [N_2222_][NTf_2_], to study the redox properties of mesoporous graphene nanoflakes, showing a capacitance above 100 F/g, while M. Pajaket al. [64], have demonstrated that some protic ammonium based ILs with nitrate anion (ethylammonium nitrate and propylammonium nitrate) can act as proton donors in the electrosorption of H_2_ in palladium. This result is quite relevant because most of the “first generation” ionic liquids were discarded over the years.

Pyrrolidinium-based ionic liquids are considered among the most promising electrolytes for the development of novel and sustainable portable energy devices. Therefore, very recently, a significant increase on the reported electrochemical studies in such ILs was observed.

In 2016, Bouvet and Krautscheid [65] verified that the redox potentials of a Fe(II)/Fe(III) system using pyrrolidinium-based ILs with L-proline and ferrocene as building blocks are independent on the IL anion and on the alkyl chain length.

After, a series of pyrrolidinium-based ILs (with different cation chain lengths) bearing the [NTf_2_]^−^ anion has been used for the electrochemical synthesis of 2–3 nm Pt nanoparticles [66]. The electrochemical reduction of [Pt(acac)_2_] in the aprotic pyrrolidinium-based ILs, 1-R-1-methylpyrrolidinium bis(trifluoromethylsulfonyl)amide (R = butyl, hexyl or decyl) was characterized by cyclic voltammetry and suggested that the reduction of [Pt(acac)_2_] to metallic platinum occurred through a two-electron transfer process without the formation of any intermediate species.

**Figure 15:**
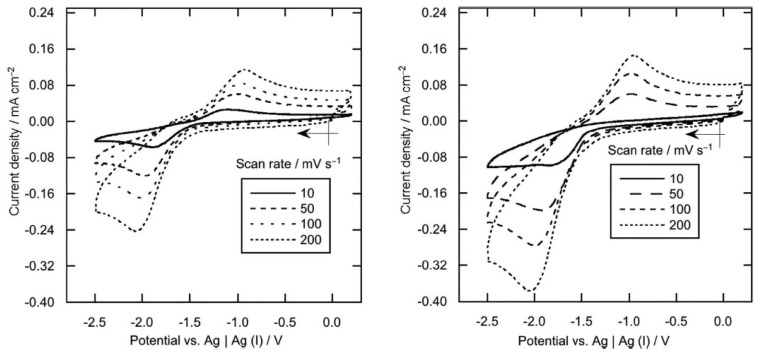
Cyclic voltammetry of [Sm(NTf_2_)_3_] (10 mM) in [bmpy][NTf_2_] at a glassy-carbon electrode at various scan rates, 25 °C (**left**) and 100 °C (**right**). Reproduced with permission from [67], copyright 2019, ECS.

M. Manjun et al. [67], explored the electrochemical reaction of samarium species in 1-butyl-1-methylpyrrolidinium bis(trifluoromethylsulfonyl)amide, [bmpy][NTf_2_], at different temperatures. The Sm system was electrochemically active, exhibiting a cathodic peak corresponding to the Sm(III)/Sm(II) reduction and an anodic one corresponding to the Sm(II) oxidation formed during the prior cathodic scan (Figure 15).

The cathodic peak current density at 100 °C was higher than that at 25 °C, reflecting a decrease in the IL viscosity at 100 °C. Moreover, the disappearance of the anodic current peak at 100 °C was observed and considered to be caused by a chemical reaction of Sm(II) in the ionic liquid. The proportionation and disproportionation equilibrium among Sm, Sm(II), and Sm(III) also led to the formation of Sm nanoparticles in the ionic liquid at 100 °C.

An IL with the same cation but with [DCA]^−^ (DCA = dicyanamide) anion was used in a hybrid electrolyte containing a redox additive, improving the specific capacitance of N-doped reduced graphene aerogel capacitors [68]. The electrochemical reduction mechanism of NbF_5_ and NbCl_5_ in the ionic liquid 1-butyl-1-methylpyrrolidinium trifluoromethanesulfonate, [bmpy][OTf], was also reported very recently [69].

Lahiri et al. [70] synthesised germanium-tin alloys with two different ionic liquids, 1-butyl-1-methylpyrrolidinium trifluoromethylsulfonate ([Py_1,4_]TfO) and 1-butyl-1- methylpyrrolidinium bis(trifluoromethylsulfonyl)amide ([Py1,4]Tf_2_N). The authors performed voltammetric studies of both ionic liquids and conclude that the best ionic liquid to be used as an electrolyte, to prepare Ge_1-x_S_nx_, is [Py_1,4_]TfO. Cyclic voltametric experiments run with the precursors (SnCl_2_ and GeCl_4_) allowed for the detection of a peak associated to the formation of the germanium-tin alloys.

Gligor et al. [71] prepared and characterized new films based on poly-3,4-ethylenedioxythiophene (PEDOT) for nitrite detection on two substrate materials (glassy carbon and gold) and four electrolytes (water, acetonitrile, 1-butyl-1-methylpyrrolidinium bis(trifluoromethylsulfonyl)imide and 1-ethyl-3-methylimidazolium bis(trifluoromethylsulfonyl)amide). The authors observed that the electrodes prepared in ionic liquids led to higher electrocatalytic activity and better sensitivities.

Phosphonium cation-based ILs are a readily available family of ionic liquids that, for some applications, offer superior properties as compared to nitrogen cation-based ILs. Recent applications cover their use as extraction and synthetic solvents, as well as in electrochemical processes, such as corrosion protection or electrolytes in batteries and super capacitors. Relevant recent examples follow.

**Figure 16:**
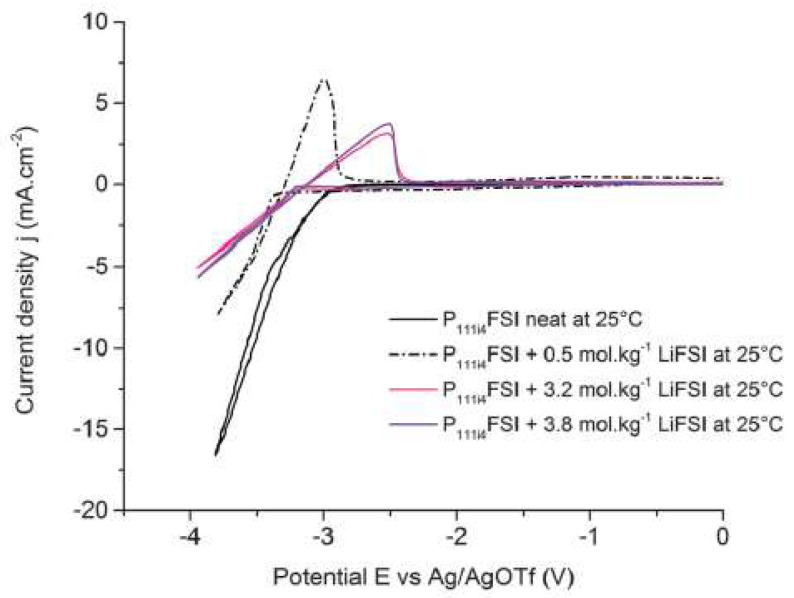
Linear sweep voltammograms (1st cycle) for neat [P_111i4_][ NTf_2_] and solutions of 0.5, 3.2 and 3.8 mol kg^−1^ Li[NTf_2_] in [P_111i4_][ NTf_2_] at a Ni working electrode with a scan rate of 20 mV s^−1^ at 25 °C. Reproduced with permission from [72], copyright 2015, RSC.

Girard et al. [72], studied the electrochemical performance of trimethyl(isobutyl)phosphonium bisfluorosulfonyl)imide, [P_111i4_][NTf_2_], by varying the concentration of Li[NTf_2_]. The results are presented in Figure 16. The voltammograms indicate a quasi-reversible Li deposition–dissolution process. A small shift of the onset potential towards fewer negative values was observed with the increase in lithium-ion concentration in accord with the Nernst equation. The oxidation current densities for the reversible peak decreased significantly with increasing lithium salt concentration as expected from the transport property trends. The authors concluded that the solutions of Li[NTf_2_] in the [P_111i4_][NTf_2_] IL exhibited reasonable transport properties, and therefore could be promising electrolytes for lithium batteries.

Khrizanforov et al. [73] applied the phosphonium-RTIL dodecyl(tri-tert-butyl)phosphonium tetrafluoroborate, [(t-Bu)_3_PH][BF_4_], in new carbon paste electrode to study the redox properties of an iron complex ({μ^2^-[Fe^II^(η^5^-C_5_H_4_–P(PhOO)(η^5^-C_5_H_4_–P(PhOOH))]_3_Fe^III^}·THF). The authors could conclude that the prepared electrode shows high conductivity, a large electrochemical window (5.6 V), stability in time, and reproducibility. The cyclic voltammetric analysis of the iron complex using the new carbon paste electrode ionic liquid confirmed two different iron redox centers in oxidation states (II) and (III). Also, the electrodeposition of Nd(III) in triethylpentylphosphonium bis(trifluoromethanesulfonyl)imide, [P_2225_][NTf_2_], at high temperature performed by Ota et al. [74] was confirmed by the cathodic peak detected by cyclic voltammetry.

**Figure 17:**
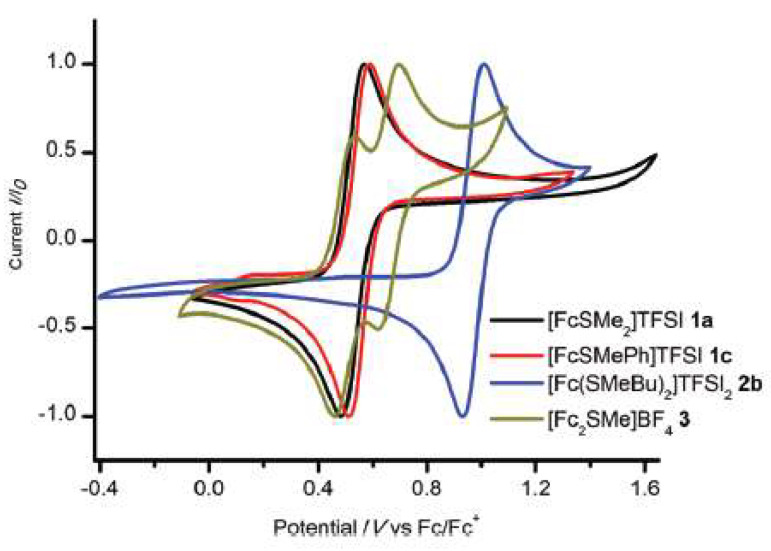
Cyclic voltammograms of selected new ferrocenyl sulfonium compounds plotted against Fc/Fc+. The measurements were performed in [emim][NTf_2_] with an Ag/Ag NTf_2_ reference electrode. Reproduced with permission from [75], copyright 2018, RSC.

Sulfonium-based ILs, namely ferrocenyl-sulfonium ILs were applied by Venkeret et al. [75]. to investigate the effect of the sulphur atom substituent in redox behaviour of the ionic liquid. They reported that electron withdrawing substituents cause a positive shift of the Fe(II)/Fe(III) ferrocene redox potential (Figure 17). The study concluded that this class of room temperature ionic liquids might be useful as redox mediators for dye-sensitized solar cells (DSSCs), as redox electrolytes in supercapacitors, or as overcharge protection additives in batteries.

**Figure 18:**
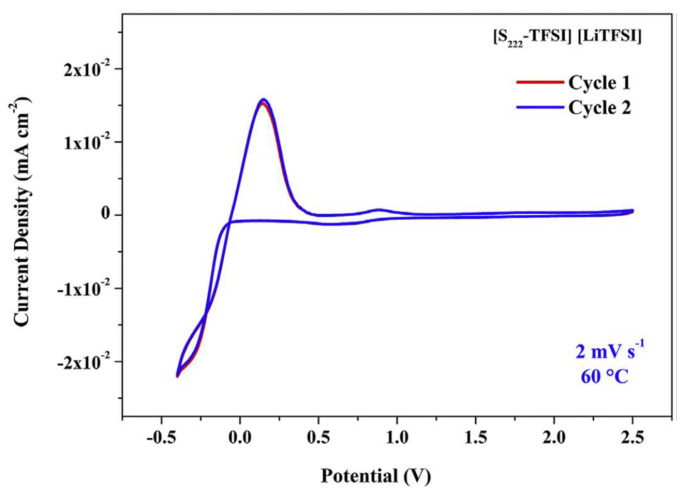
Cyclic voltammetric measurements of the [S_222_][NTf_2_]-LiNTf_2_ IL electrolyte. Reproduced with permission from [76], copyright 2019, Elsevier.

The redox properties of diethylmethylsulfonium bis(trifluoromethylsulfonyl)imide, [S_222_][NTf_2_], together with different lithium salts were electrochemically characterized by Rangasamy [76]. The system [S_222_][NTf_2_]-LiNTf_2_ appears stable in a wide potential range and exhibits a reversible redox behaviour (Figure 18), which are excellent features to be applied as an electrolyte for practical battery applications.

Figure 18 shows the cyclic voltammetry of the [S_222_][NTf_2_]-LiNTf_2_ ionic liquid electrolyte measured at 60 °C. As reported by the authors, both forward and reverse scan redox peaks have been observed in both cycles. One is the lithium deposition process, and the other is related to the lithium stripping process. The current density of the both the peaks are in the same range indicating that the ionic liquid electrolyte exhibits a good reversible redox behaviour. This more evidence of the potential role that this ionic liquid electrolyte could have as a potential candidate for a “workable” electrolyte to be used in a lithium cell. A wide electrochemical stability window is one of the most desired properties of an “ideal” electrolyte because the chemistry of the two electrode-electrolyte interfaces involved in the battery depends on the properties of the electrolyte. Usually, it is the oxidative stability of the anion that determines the anodic limit of the ILs. Parameters such as being a weak Lewis acid result in weak interactions with the weakly Lewis-acidic organic cations in the liquids, making them have relatively good anodic stability. Because such cations and anions have difficulty to discharge on the electrodes, mainly at lower potentials, the result is a large window of the electrochemical stability.

**Figure 19:**
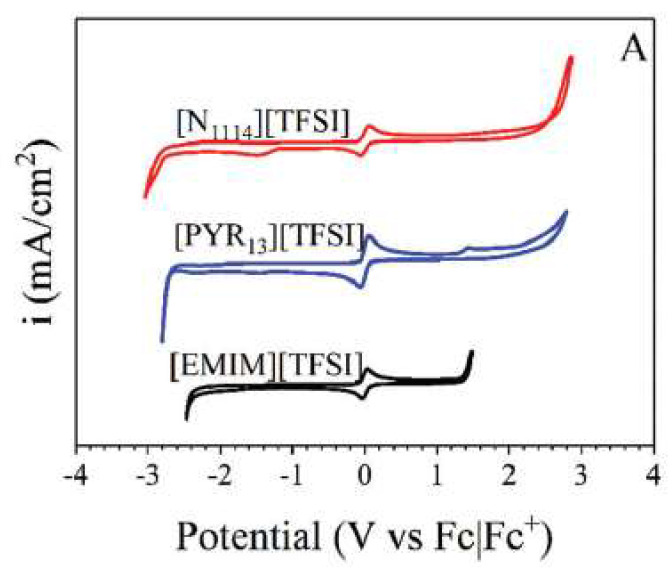
Cyclic voltammograms of [emim][NTf_2_], [N_1114_][NTf_2_], and [Pyr_13_][NTf_2_] with 10 mM ferrocene (Fc). Scan rate = 20 mV/s. Reproduced with permission from [77], copyright 2018, RSC.

Klein et al. [77] selected different families of ILs (ammonium, imidazolium and pyrrolidinium) to measure their differential capacitances. Thus, butyl-trimethylammonium bis(trifluoromethyl sulfonyl)imide, [N_1114_][NTf_2_], ethyl-methylimidazolium bis(trifluoromethylsulfonyl)imide, [emim][NTf_2_], and methyl-propylpyrrolidinium bis(trifluoromethylsulfonyl)imide, [Pyr_13_][NTf_2_], were analysed by electrochemical impedance spectroscopy (EIS) over the entire electrochemical window determined by cyclic voltammetry (with Fc|Fc^+^ internal reference, as shown in Figure 19).

In this work, the quaternary ammonium-based IL displayed a different electrochemical potential window and the difference to the previous publications was attributed by the authors [77] to a difference in water content, trace impurities, or a difference in current cut off. One of the most important conclusions from this work is related to the factors for the increase in capacitance. It was expected that [N_1114_] would display crowding behaviour at the interface while [Pyr_13_] and [emim] formed an interface structure with an over-screened surface. These findings support the fact that the bulk measure of molecular interactions, such as viscosity, do not influence the behaviour of the IL at the nanoscale lengths of the electrode–electrolyte interface.

Vélez applied dicationic pyrrolidinium and piperidinium ILs with [NTf_2_]^-^ anion as electrolytes in lithium batteries [78]. Later, a list of imidazolium-, pyridinium-, pyrrolidinium-, and piperidinium-based ILs was used together with a carbon paste electrode to study the electrochemical response of dopamine [79], and the results showed that the sizes and types of both cation and anion have an influence on that response.

Overall, all the reported IL classes can be used in a large number of electrochemical applications with promising results and their usage as electrolytes or electrolyte additives is expanding this area. In the future, other ILs families should be tested, namely guanidinium-based ILs.

## 4. General Remarks Considering ILs Purification and Viscosity

From the above-mentioned published contributions, it is clear that impurities, such as water content or contamination with halides, can lead to significant changes in the expected electrochemical results. Therefore, purifying the ILs is a basic requirement to obtain reliable cyclic voltammograms. Several technologies, i.e., adsorption, aqueous two-phase extraction, crystallization, distillation, extraction, membrane separation, and external force field separation, have been investigated for the recovery and purification of ILs either in the lab or at pilot scale.

A small survey of the techniques in use nowadays (Table 4) ilustrates the plethora of possibilities regarding the recovery and purification of ILs.

Several solvents, such as water, organic solvents, and supercritical carbon dioxide (scCO_2_), have been tested for ILs extraction processes. Aqueous two-phase extraction (ATPE) is used when two immiscible phases (both soluble in water), e.g., polymer/polymer, polymer/salt, or salt/salt, are placed into contact with each other above the critical concentration at a specific temperature. This type of extraction is recognized since it is a rapid, low-cost, and scalable technology for separation and purification of antibiotics, enzymes, therapeutic proteins, etc. In this method, the ILs can be concentrated and recovered in the IL-rich phase.

The employment of gravity field, as well as centrifugation and/or magnetic fields have been proposed for recovery of hydrophobic ILs or solutions of magnetic ILs [80].

Recently, the use of membrane processes for the recovery and purification of ILs, taking advantage of the selective permeability of the membranes, has been employed. One of its features is the relatively low energy consumption and simple operation procedures required.

Table 5 ilustrates the advantages and disadvantages of the above methods.

Another factor to be considered is the viscosity of ILs. They are more viscous than common organic solvents, and therefore exhibit a diferent behaviour when submitted to a potential.

Recently, Margulis et al. [80] classified ILs structure in three possible structural motifs associated with (i) vicinal interactions, (ii) formation of positive–negative charge-alternating chains or networks, and (iii) alternation of these networks with apolar domains. They concluded that friction and mobility of Ils are nowhere close of being spatially homogeneous, and called “mechanical heterogeneity” to this phenomem, where charge networks are intrinsically stiff and charge-depleted regions are softer, flexible, and mobile. Moreover, they proposed that charge blurring associated with the loss of memory of where charges (positive and negative) are within networks is the key mechanism associated with viscosity in ILs. An IL will have low viscosity if a characteristic charge-blurring decorrelation time is low [81]. Lower viscosity implies higher conductivity and more efficient mass transport for the applications of electrochemical systems. The anion is responsible for viscosity, so its proper selection would have advantageous implications in the system environment where transport properties are pivotal. Therefore, the physicochemical properties and structure–property relationships of the ionic species in ILs, especially the effects of cationic structures on the transport properties, including viscosity, conductivity, and electrochemical properties, are still in need of more studies.

## 5. Conclusions

There are endless combinations of mixed salts that can be designed on demand. Already, novel ionic liquids are being vigorously developed by the scientific community, but the next step should be the fine-tuning of their functionalities, e.g., by mixing two or more ionic liquids. Even with all of the synthetic advances, one of the biggest limitations to the use of RTILs is their cost. Common RTILs remain quite expensive, particularly when compared to conventional organic solvents. For example, the price of one liter of THF is ca. $60, whereas butylmethylimidazolium tetrafluoroborate ([bmim][BF_4_], one of the least expensive RTILs at the moment) is over $2000 per liter [82]. As a result, a very important research area is the design of much less expensive, equally stable ILs. Several examples presented in this work mention the economic impact or the potential aspects of producing an ionic liquid that could effectively extract, e.g., rare earth elements.

Over the years, ionic liquids have exhibited excellent properties as electrolytes, not only because of the absence of an organic solvent, but also due to their electrochemical stability, which allow them to be used in a wide electrochemical potential window without being oxidized or reduced. In addition, they allow for the study of the redox behaviour of organic and inorganic compounds by a simple technique such as cyclic voltammetry.

ILs can also be used as additives, as well as mixed with inorganic salts, which has also presented promising results. In this contribution, several examples were given whereby the role of the IL to dissolve or to crystalize materials can be used for energy or sensing applications, making them a brand-new field to discover.

It is expected that the electrochemical characteristics of ionic liquids will continue to find meaningful applications. Several examples were given in the production of new materials or methods. They show physicochemical properties, such as polarity, basicity, acidity, solution temperature, and even reaction step by a colour change. A conductive liquid can transport not only ions but also electrons, microchips, and so on. A liquid battery can be prepared by coating three layers of electrodes and electrolytes.

In general, the electrochemical field in ILs is gaining space in chemical, biological, and energy areas, and it deserves to be explored further. The ILs are offering significant advantages over the conventional solvents, but due to the huge number of ILs and parameters to be optimized, more tests should be done.

Most of the published work presented in this review are ground-breaking articles, such that now more contributions are required to be able to develop new fields in chemistry. With advances in the science of ionic liquids, there are bound to be some excellent treatments for ionic liquid decomposition. Ionic liquids could be developed to have electrolytes that carry a decomposition switch. Both developments would make ionic liquids more “green” and useful electrolyte materials. The recovery and recycling processes and other surrounding technologies should be developed along with the progress made in the functional design of ionic liquids.

It is also important to mention that the complexity of most chemical reactions or separation systems makes the use of a single method unable to reach the required purity for ILs electrochemical studies. To improve the purification efficiency, the conjugation of several techniques may be needed.

## Figures and Tables

**Figure 1:**
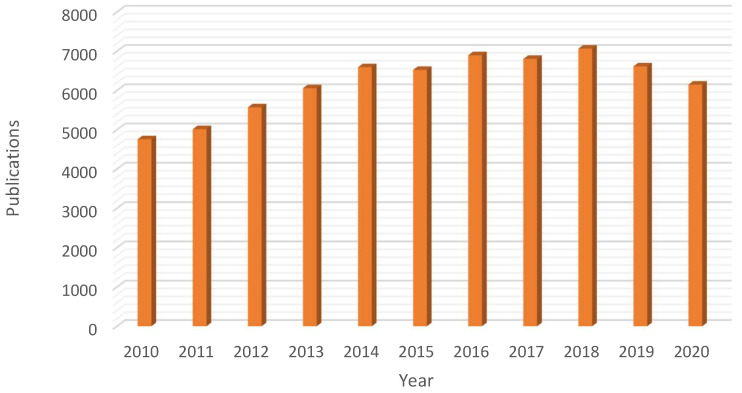
Rising interest on the use of ionic liquids: yearly number of publications in the domain of ionic liquids in the 2010–2020 period (Database: Scopus, search terms: “ionic” AND “liquids”, search date: 7 November 2020).

**Table 1:** General characteristics of room temperature ionic liquids [7].

General Properties	Features
Low melting point Non volatility Composed by ions Organic ions	• Treated as liquid at ambient temperature • Wide temperature interval for applications • Thermal stability • Flame retardancy • High ion density • High ion conductivity • Designable/Tuneable • Unlimited combinations possible

**Table 2:** Structures of most common ions used in designing ionic liquids. Adapted, with permission of ref. [5], copyright, 2020, Elsevier.

**Cation**	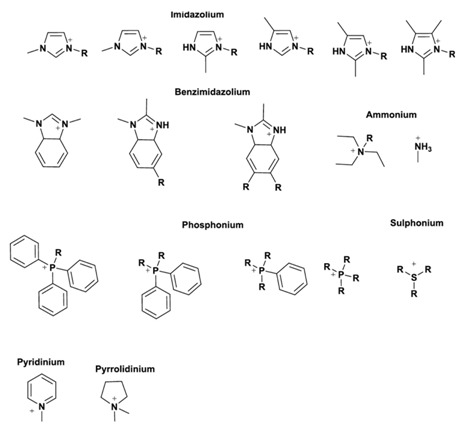
Anion	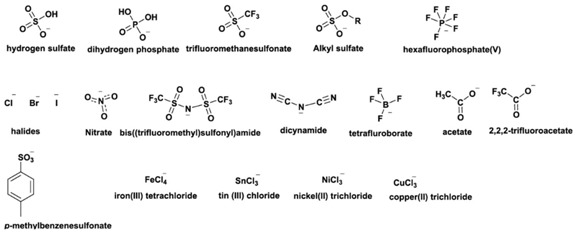

**Table 4:** Comparison of ionic liquids recovery methods. Adapted with permission from [80], copyright 2018, RSC.

Methods	Characteristics	
Distillation	Distillation of volatile compounds	ILs are remained as residue.
	Distillation through reactions of ILs	ILs are distilled as neutral species or intact ion pairs.
Extraction	Extraction with water	Extract hydrophilic solutes from hydrophobic ILs.
	Extraction with organic solvents	Extract hydrophobic solutes from ILs.
	Extraction with scCO_2_	Both hydrophobic and hydrophilic ILs can be separated.
Adsorption	Adsorption by ACs	Affected by pore structure and surface chemistry of ACs.
	Adsorption by soils and sediments	Affected by TOC and CEC of soils.
	AAdsorption by ion exchange resins	Affected by functional group and ionic form of resins.
Membrane separation	Pressure-driven membrane techniques	ILs can be either permeated or rejected.
	Pervaporation	ILs are rejected while volatile species are permeated.
	Membrane distillation	ILs are rejected while water vapor is permeated.
	Electrodialysis	Cations and anions of ILs cross ion exchange membranes.
Aqueous two-phase extraction (ATPE)	ATPE based on chemicals addition	Formation of ATPS by adding salts, carbohydrates or CO_2_.
	ATPE based on changing temperature	Formation of ATPS or LCST-type phase separation.
Crystallization	Solution crystallization	ILs are crystallized from solution.
	Melt crystallization	ILs are crystallized from melt.
	Pressure-induced crystallization	ILs are crystallized under high pressure.
Force field	Gravity separation	ILs are separated from immiscible liquids.
	Centrifugation	ILs emulsion were separated by centrifugation.
	Magnetic separation	ILs are separated by magnetic field.

**Table 5:** Comparison of the advantages and disadvantages for the purification methods in ionic liquids. Adapted with permission from [80], copyright 2018, RSC.

Methods	Advantages	Disadvantages
Distillation	Simple to operate	Energy consuming
		Low impurity
Extraction	Simple, low cost	Limited application
		Cross-contamination
		Requirement of special apparatus
Adsorption	Non-destructive, suitable for diluted solutions	Insufficient desorption data
Membrane separation	Low energy demand, selective permeability	Concentration polarization
		Large membrane area
		Membrane fouling
		Membrane fouling
Aqueous two-phase extraction	Rapid, low-cost, scalable	High concentration of salts or organics
		Limited application
Crystallization	High purity	Energy consuming
Force field	Simple, low energy demand	Low separation rate
		Small throughput
		Limited application
